# *ExDoRA*: enhancing the transferability of large language models for depression detection using free-text explanations

**DOI:** 10.3389/frai.2025.1564828

**Published:** 2025-05-21

**Authors:** Y. H. P. P. Priyadarshana, Zilu Liang, Ian Piumarta

**Affiliations:** Kyoto University of Advanced Science (KUAS), Kyoto, Japan

**Keywords:** LLM transferability, in-context learning, free-text explanations, prompt engineering, digital mental health, natural language processing

## Abstract

Few-shot prompting in large language models (LLMs) significantly improves performance across various tasks, including both in-domain and previously unseen natural language tasks, by learning from limited in-context examples. How these examples enhance transferability and contribute to achieving state-of-the-art (SOTA) performance in downstream tasks remains unclear. To address this, we propose *ExDoRA*, a novel LLM transferability framework designed to clarify the selection of the most relevant examples using synthetic free-text explanations. Our novel hybrid method ranks LLM-generated explanations by selecting the most semantically relevant examples closest to the input query while balancing diversity. The top-ranked explanations, along with few-shot examples, are then used to enhance LLMs’ knowledge transfer in multi-party conversational modeling for previously unseen depression detection tasks. Evaluations using the IMHI corpus demonstrate that *ExDoRA* consistently produces high-quality free-text explanations. Extensive experiments on depression detection tasks, including depressed utterance classification (DUC) and depressed speaker identification (DSI), show that *ExDoRA* achieves SOTA performance. The evaluation results indicate significant improvements, with up to 20.59% in recall for DUC and 21.58% in F1 scores for DSI, using 5-shot examples with top-ranked explanations in the RSDD and eRisk 18 T2 corpora. These findings underscore *ExDoRA*’s potential as an effective screening tool for digital mental health applications.

## 1 Introduction

Few-shot prompting of large language models (LLMs), which involves learning from a small number of in-context examples within prompts, has led to significant improvements across various natural language processing (NLP) tasks, including classification, generation, multi-step reasoning, and summarization (Brown et al., 2020; Chowdhery et al., 2023). These in-context examples, also called demonstrations, cast downstream tasks together with task-specific prompts into a frozen LLM format to achieve state-of-the-art (SOTA) in-context learning (ICL) performance for both in-domain, contextually similar tasks and previously unseen, contextually dissimilar ones (Qin et al., 2022; Gao et al., 2021; Li et al., 2023). However, the quality of the retrieved demonstrations and how they contribute to SOTA ICL downstream performance remain unclear. Free-text explanations, on the other hand, have received increasing attention by providing detailed reasoning behind an LLM’s decisions over extractive methods such as SHapley Additive exPlanations (SHAP) Local Interpretable Model Agnostic Explanation (LIME), which focus on input tokens (Wiegreffe et al., 2022). Inspired by the critical role that explanations play in human learning to adapt knowledge to new tasks (Ahn et al., 1992), there is a pressing need to enhance the quality and consistency of demonstrations in ICL, thereby improving the downstream performance of previously unseen tasks through the most suitable free-text explanations (Lampinen et al., 2022).

Linguistic-based detection of depression on social media offers notable benefits over clinical and vision-based methods, particularly in early identification by analyzing shifts in language patterns, mood, or behavior (Le-Hinh et al., 2023). Detecting depressive language in social media posts using a model trained on social media text data tagged for depressive symptoms is considered an in-domain task. Multi-party conversations (MPCs), on the other hand, involve a wide range of language use, including emotions, thoughts, and social interactions, making them crucial for detecting depression as a complex, contextually dissimilar task (Lu et al., 2023). While it is feasible to use top-ranked demonstrations for depression detection in MPCs, the reasoning behind the model’s outcome remains uncertain. Improving an LLM itself to understand previously unseen depression detection in MPCs as an ICL downstream task using free-text explanations is currently unexplored.

In this article, we propose *ExDoRA*, a novel LLM transferability framework designed to elucidate the most appropriate demonstrations using synthetic free-text explanations generated by multiple LLMs, including Mistral-7B-Instruct, which is known for reliable explanation generation in emotion discovery (Siino, 2024), to improve the quality of demonstrations for depression detection tasks. Our objective is to enhance LLM knowledge transfer in MPC structure and semantic modeling for previously unseen depression detection tasks by utilizing a *reason-then-predict* approach (Ye and Durrett, 2022). We evaluate the factuality of generated explanations by examining their alignment with the intended context and assess their consistency by analyzing the impact of these explanations on the final prediction.

**Figure 1:**
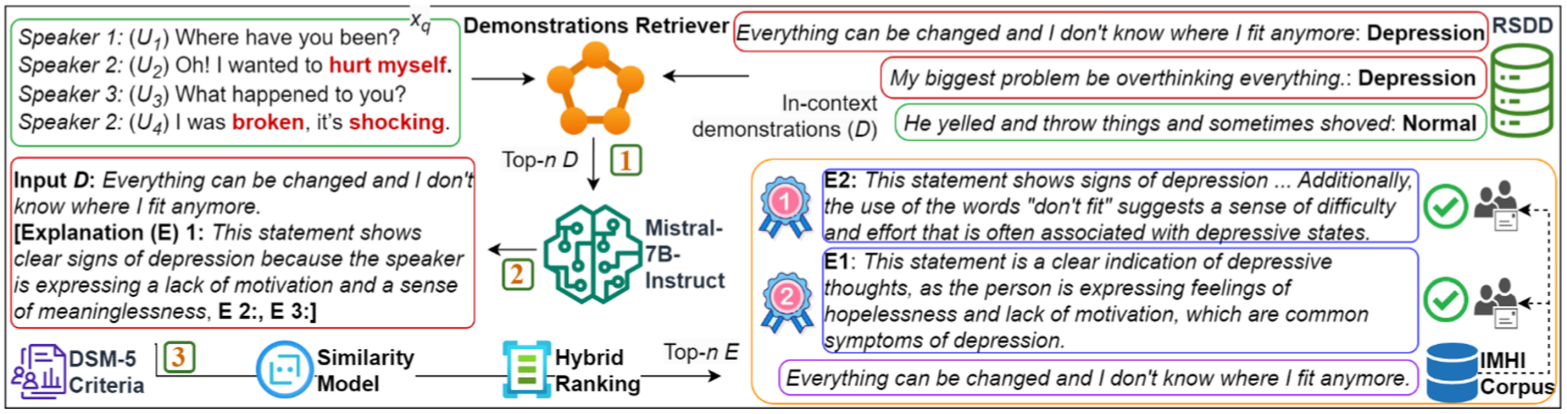
The proposed *ExDoRA* will select top-ranked explanations for depression detection in text-based MPCs.

As shown in Figure 1, *ExDoRA* comprises three key phases. First, the demonstration retriever selects the most semantically relevant demonstrations from a depression corpus closest to the input MPC query. Next, the top-ranked demonstrations are used to generate free-text explanations for the query. Finally, these generated explanations are ranked to identify the best ones by validating them externally using the interpretable mental health instruction (IMHI) corpus (Yang et al., 2024). The selected demonstrations and explanations are then used for few-shot prompting, employing soft prompt templates and soft verbalizers specifically designed to support the core logic of the prompt manager for MPC modeling knowledge transfer, enabling the classification of depressed utterances and the identification of depressed speakers. Our main contributions include:

1 Designing a novel framework for selecting top-ranked explanations through a hybrid selection strategy that combines expected reciprocal rank (ERR) (Chapelle et al., 2009) and maximum marginal relevance (MMR) (Carbonell and Goldstein, 1998). ERR considers the probability of a user finding a relevant demonstration at each rank position, which can be used to prioritize the most relevant explanations efficiently. MMR, on the other hand, balances relevance with diversity, aiming to ensure that the selected explanations are not only relevant but also cover different aspects of the content, including context shifts of MPCs. To the best of our knowledge, we are the first to ensure the selection of the most relevant explanations for out-of-domain (OOD) tasks, both for demonstrations and the input query, while promoting diversity in the outputs to prevent redundancy.
2 Evaluating the factuality and consistency of the synthetic free-text explanations using the IMHI corpus.
3 Employing multiple downstream depression detection tasks to evaluate the generalization of our methods, incorporating *ExDoRA* as a component into the ProDepDet framework (Priyadarshana et al., 2024) established for OOD knowledge transfer.
4 Conducting ablation studies to evaluate the robustness of the proposed framework concerning the number, order, and diversity of the top-ranked explanations.

The overall organization of this article is as follows: Section 2 reviews related work and Section 3 elaborates the proposed architecture and methodology. The experiments are presented in Section 4 and then discussed in Section 5.

## 2 Related work

### 2.1 Free-text explanation generation

The generation of suitable free-text explanations plays a crucial role in enabling few-shot demonstrations in previously unseen depression detection tasks. Research has shown that combining few-shot demonstrations with appropriate explanations improves downstream performance across multiple in-domain tasks. The earliest neural models for generating free-text explanations were developed for computer vision tasks (Hendricks et al., 2016; Kim et al., 2018) and natural language inference (NLI) (Camburu et al., 2018), relying on supervised corpora. Rajani et al. (2019) and Shwartz et al. (2020) further advanced these methods by enhancing both supervised and unsupervised approaches to improve the performance of in-domain question and answering (QA) downstream tasks. Wiegreffe et al. (2021) were the first to introduce a pipeline for generating free-text natural language explanations to improve reasoning rather than solely focusing on task-specific performance. Paranjape et al. (2021) and Marasović et al. (2022) utilized prompt engineering techniques over LLMs to generate explanations for commonsense reasoning tasks using human-written explanations, but the downstream performance fell short of expectation. Inspired by previous work on human-assisted few-shot LLM explanation generation, studies such as those by Sun et al. (2022), Ye and Durrett (2022), and Wiegreffe et al. (2022) explored QA and NLI tasks, while Wang et al. (n.d.) introduced a counterfactual reasoning framework for generating choice-specific explanations in multiple-choice QA, although this was limited to contextually similar tasks. To address the challenge of poor generalization to unseen tasks, Ludan et al. (2023) used non-human-generated free-text explanations from LLMs for classification tasks, although this approach was limited to unseen scenarios within the same domain. Liu et al. (2024) recently introduced a free-text explanations-based interpretability framework that achieved SOTA performance for QA tasks. However, its performance declined significantly when applied to OOD unseen scenarios. While various studies have focused on improving in-domain downstream tasks using free-text explanations, the transfer of knowledge to OOD tasks remains an area for further exploration.

### 2.2 Linguistic-based depression detection

Due to the limitations of clinical diagnosis, linguistic-based depression detection on social media data has emerged as a rapidly evolving research area. The initial effort to uncover the link between natural language use and depression detection was made in 2017 (Losada et al., 2017), followed by early exploration of linguistic patterns for identifying depression (Burdisso et al., 2019). A few machine learning-based approaches, such as those by Burdisso et al. (2021) and the DEPTWEET model by Kabir et al. (2023), have contributed to improving depression identification by leveraging linguistic patterns. To overcome the limitations of these earlier methods, neural-based techniques were developed, such as the ordinal classification technique introduced by Naseem et al. (2022) for early depression detection and a recurrent neural network-based method by Ghosh and Anwar (2021) that estimates depression severity using self-supervised data. These methods were further enhanced by ICL LLM-based approaches, including text summarization-based depression detection (Zogan et al., 2021), mental health prediction tasks (Xu et al., 2024), multi-modal tasks (Sharma et al., 2024), and explainable LLM-augmented chatbots (Liu et al., 2023). However, these methods failed to gain end-user trust due to concerns regarding the explainability of their outcomes. To improve the explainability of black-box depression detection models, several strategies have been proposed. These include a text-to-text explainable pipeline (Bao et al., 2024), integration of LIME and SHAP extractive explanations (Malhotra and Jindal, 2024), treating mental health analysis as a text-generation task (Liu et al., 2023), and human-assisted prompt-based explanation generation as a predict-then-explain approach (Qin et al., 2023). Despite these advances, none has demonstrated satisfactory performance in contextually dissimilar cases, such as depression detection in MPC analysis using task-specific few-shot demonstrations and free-text explanations.

## 3 Methods

### 3.1 Approach

Our aim is to model previously unseen depression detection as an OOD task by leveraging an LLM’s knowledge of MPC modeling. This involves incorporating carefully selected demonstrations and their corresponding free-text explanations. As shown in Figure 1, the demonstration retriever *Dr* is responsible for retrieving the most relevant top-ranked demonstrations, *D* = {*d_1_*, *d_2_*, …, *d_n_*}, for the input MPC utterance *xq*, where (*xq*, *yq*) ∈ *D* is a pair of MPC utterance and its ground truth, sourced from the expert-annotated Reddit Self-reported Depression Diagnosis (RSDD) corpus (Yates et al., 2017). Given its effectiveness in retrieving demonstrations from unseen datasets across multiple ICL tasks, we selected a unified demonstration retriever (UDR) (Li et al., 2023) as our primary retrieval mechanism. These demonstrations are then used to generate explanations *E*, considering both *D* and each utterance in *xq,* with the help of multiple foundational LLMs, including Mistral-7B-Instruct. The generated *E* is ranked, validated, and then paired with MPC source prompt embeddings *P* = {*p_1_*, …, *p_k_*} and *D* for OOD depression detection. In the following sections, we present the design, *E* generation, *E* ranking, *E* validation, the components of the prompt manager, and the formation of the depression detection tasks.

### 3.2 System design

**Figure 2:**
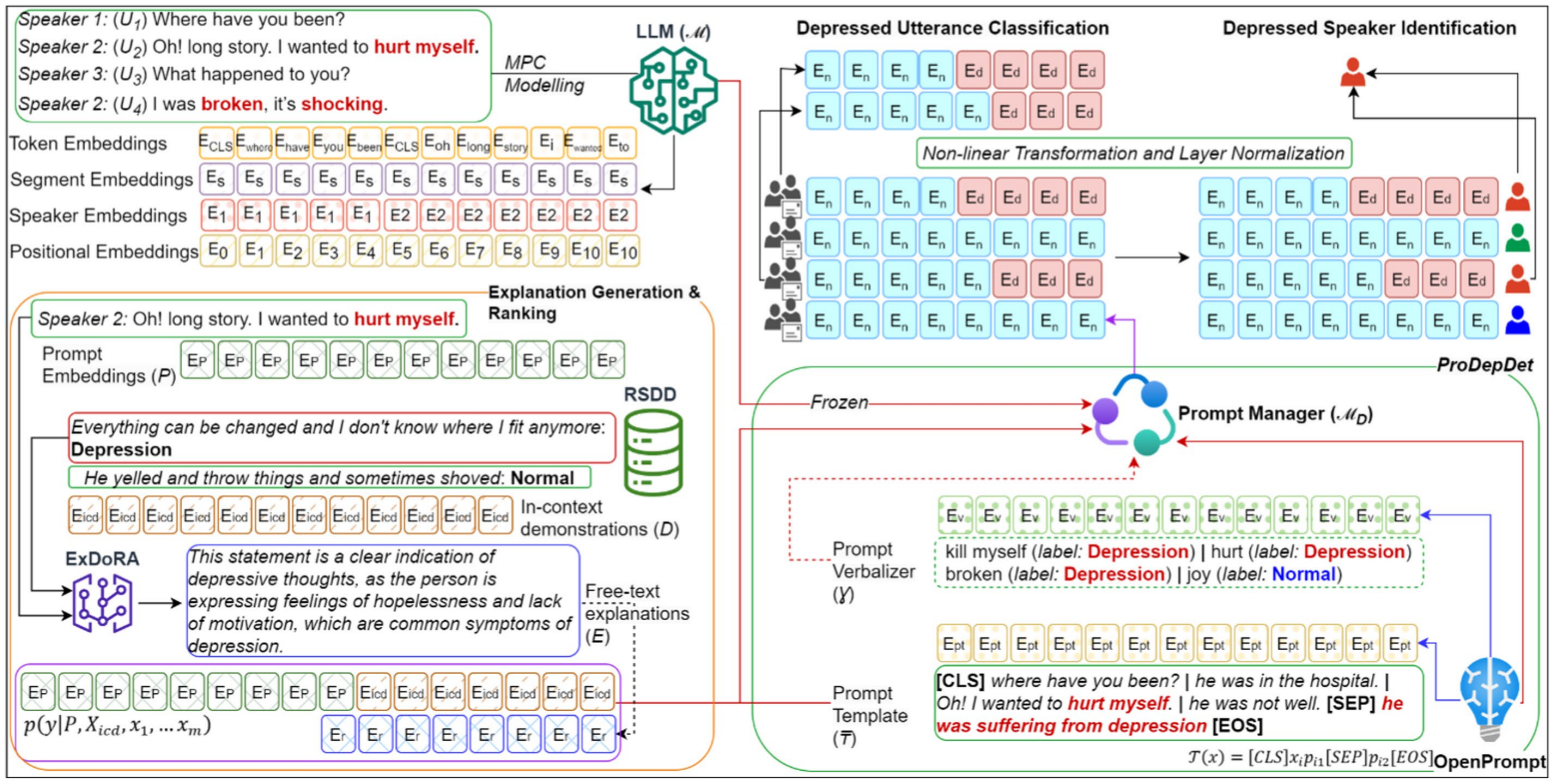
The system design for transferability of LLMs for depression detection using *ExDoRA*.

Figure 2 shows the design of the proposed system. At its core, we employ a pre-trained LLM capable of modeling MPCs as the “*frozen*” LLM. This LLM has acquired knowledge in processing contextualized representations of MPCs, including token embeddings, segment embeddings, speaker embeddings, and positional embeddings, to model MPC behaviors such as response utterance selection and exact speaker identification. Following the approach by Lester et al. (2021), most parameters of the LLM are kept unchanged, with only minor adjustments made to train the prepended embeddings for MPC modeling within *P* to detect depression. Our objective is to generalize a specific LLM to handle multiple tasks rather than creating separate instances for each task. Once the prompt embeddings *P* are paired with the demonstrations *D* and the corresponding *E* from *ExDoRA*, the prompt manager ℳ*_ꟷ_* of ProDepDet processes the embedded *P* using mandatory soft prompt templates *₸* and optional soft verbalizers *Ɣ* created from the OpenPrompt Python library. Inspired by Su et al. (2022) on the transferability of soft prompts for in-domain tasks, we empirically investigate the ICL-based transferability of these soft prompts and verbalizers for OOD tasks. These components use the frozen LLM ℳ to determine the contextualized representations for downstream depression detection, including depressed utterance classification and depressed speaker identification, applying non-linear transformation and layer normalization. A detailed version of Figure 2 is provided in Appendix.

### 3.3 Explanation generation

The generation of *E* is crucial for determining the most appropriate free-text explanations based on the demonstrations retrieved from UDR for each utterance (*U_1_*, *U_2_*, *U_3_*, and *U_4_*) in an MPC.


(1) $$E={\left\{e_{i}\right\}}_{i=1:K}$$

**Figure 3:**
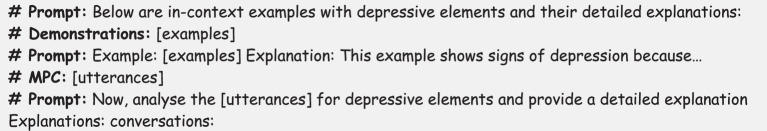
Example of a prompt used to generate free-text explanations. The prompt is designed to highlight the depressive elements within the demonstrations and input utterances, guiding the LLM to focus on recognizing and articulating relevant depressive content.

In Equation 1, each *e_i_* ∈ *Ѵ* represents a free-text explanation generated using the context vocabulary *Ѵ* of the generative LLM. In this process, we used Mistral-7B-Instruct and Gemma-7B to generate *E*. These models, trained on instruction-based data, can respond to detailed prompts by producing natural language outputs that enhance various decision support systems. Furthermore, Mistral-7B-Instruct’s instruction tuning enables it to handle structured tasks and generate explanations that are coherent and contextually relevant, enhancing an LLM’s ability to interpret and clarify subtle features, such as language patterns in depression detection. The prompt, shown in Figure 3, is adjusted to emphasize the depressive elements of both *D* and the input utterances. This helps guide the LLM in focusing on recognizing and explaining depressive content. Given the resource constraints, we limit the generation to a maximum of three explanations for each *D*.

### 3.4 Explanation ranking

The ranking of explanations *E_rank_* is conducted using a novel selection strategy that combines ERR and MMR. This approach incorporates two key components, such as depression diagnosis criteria and a similarity model. To rank the generated explanations based on their semantic relevance to *D* and the input utterances, we utilize a semantic similarity measure. One effective way to do this is by using sentence embeddings and calculating cosine similarity between the generated *E* and both *D* and the query (Ye et al., 2023). We use a pre-trained model from the *sentence-transformers* library^1^ as the similarity model ℳ*_S_* to compute sentence embeddings and then organize the explanations based on their average semantic similarity. The choice of sentence embedding models significantly impacts the effectiveness of MMR, considering the diversity of the generated explanations. Advanced models such as *all-MiniLM-L6-v2*^2^ offer robustness, which is evaluated through experiments to ensure SOTA performance.

The depression diagnostic criteria play a crucial role in ensuring that the generated explanations are clinically accurate and contextually relevant. Although our method is intended as a screening tool for depression detection, adhering to clinical guidelines is vital for ethical and responsible use in mental health contexts. To ensure the explanations reflect real-world clinical scenarios, we use DSM-5 criteria (Regier et al., 2013) as the depression diagnostic criteria. The DSM embeddings are generated using ℳ*_S_* and integrated into the same embedding space with the embeddings of *D*, *E*, and the input utterances. These contextualized representations are used to determine relevance scores for each generated explanation by evaluating the average semantic similarity. The scores are normalized to produce relevance probabilities Ꝓ_ꟷ_, which serve as input for ERR- and MMR-based ranking as presented in Algorithm 1.

**ALGORITHM 1:**
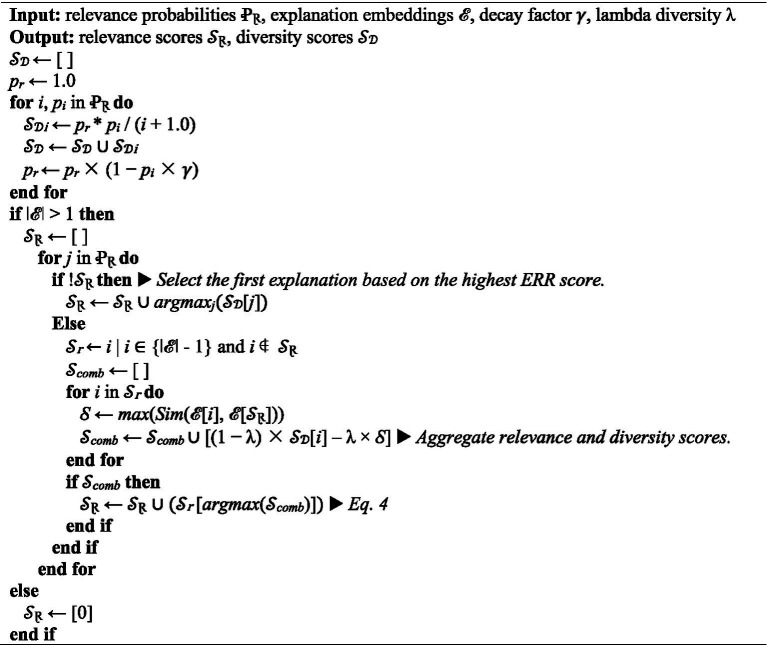
ERR and MMR-based explanations ranking.

To achieve a balance of semantic relevance and diversity in ranking the generated explanations, we use a hybrid approach combining ERR and MMR. ERR uses relevance probabilities at each rank to calculate a cumulative score as in Equation 2


(2) $$\mathrm{ERR}=\sum_{k=1}^{n}\frac{1}{k}\prod_{i=1}^{k-1}(1-{\mathrm{\gamma p}}_{i}){\mathrm{\gamma p}}_{k}$$

where *p_i_* represents the probability of relevance for the *i*-th explanation and 𝛾 denotes the decay factor (often set between 0 and 1) that simulates a user selecting the exact explanation among a few options by reducing the influence of each subsequent explanation. MMR promotes diversity by balancing the trade-off between relevance and redundancy when ranking explanations. It selects the next explanation based on both its similarity to the query *q* for relevance and its dissimilarity to already selected items *e_s_* for as Equation 3,


(3) $$\mathrm{MMR}(e_{i})=\lambda \times \mathrm{Sim}(e_{i},q)-(1-\lambda )\times (e_{i},e_{j})x_{j\in e_{s}}\text{maxSim}$$

where *e_i_* represents the candidate explanation and *λ* denotes the trade-off between relevance and redundancy. The values of Equations 2, 3 are then aggregated as *S_comb_* to determine the explanations with the highest combined scores *S_top_* as Equation 4


(4) $$S_{\mathrm{top}}=\sum_{S_{\text{comb}}}e_{s}[{\text{argmax}}_{j}(S_{\text{comb}}[j])]$$

where *argmax_j_* finds the index *j* of the maximum value in the aggregated scores.

ERR and MMR offer dynamic trade-offs between relevance and diversity, unlike alternatives such as precision at *k* and mean average precision, which focus on only one dimension (Chapelle et al., 2009; Carbonell and Goldstein, 1998).

### 3.5 Explanation validation

The top-ranked explanations are then validated using the IMHI benchmark, which serves as the evaluation corpus for mental health-related reasoning tasks. Note that we used 100 expert-written explanations from the IMHI benchmark to evaluate the generated *E*, as it is currently the only available benchmark for mental health-related reasoning tasks. The top two validated explanations are then used for few-shot prompting to facilitate depression detection. Given the input embeddings *X* = {*x_1_*, …, *x_m_*} for *m* contextual representations of *xq*, *P*, *D*, and *E_rank_*, the maximum probability of obtaining *y*, the depression ground truth corresponding to *X*, is formulated as (*y* | *P*, *D*, *E_rank_*, *x_1_*, …, *x_m_*).

### 3.6 Prompt manager

As shown Figure 2, the prompt manager ℳ*_ꟷ_* is developed using the ProDepDet framework (Priyadarshana et al., 2024). It integrates *P*, *D*, and *E_rank_* to facilitate the LLM knowledge transfer in MPC modeling for previously unseen depression detection tasks. This is achieved using the most appropriate prompt templates *₸* and soft verbalizers *Ɣ*. We employ the soft template class of the OpenPrompt library (Ding et al., 2022) to generate *₸*, leveraging its ability to encapsulate the input for ICL tasks compared to manually crafted templates. Task-specific prompt templates for depression detection tasks, such as depressed utterance classification, are derived from the MPC modeling source task *P*. For a given input utterance *x* ∈ *xq*, the template text *T* is structured as in Equation 5.


(5) $$T(x)=[\mathrm{CLS}]x⊕p_{1}[\mathrm{SEP}]p_{2}[\mathrm{EOS}]$$

The [CLS], [SEP], and [EOS] tokens are critical components in LLM ICL classification-based tasks. [CLS] is added at the beginning of *xq* to gather the overall context. [SEP] is used to separate distinct segments within *xq*, allowing it to understand the individual and combined context. [EOS] marks the end of a sequence, signaling the LLM to stop processing further tokens. Inspired by Sun et al. (2024) in ICL for contextually dissimilar tasks, *p_1_*, *p_2_* ∈ *P* are concatenated at the end of each *X* in *x* and the end of *xq*. This can be illustrated using an MPC example, “*T*(*x*) = [CLS] *where have you been?* | he was in the hospital | *Oh! I wanted to hurt myself.* | he was feeling unwell [SEP] he was experiencing symptoms of depression [EOS]” where the bold text presents samples for *T*. The length of *T* is considered a key design factor, and several ablation studies were performed to assess the impact of varying prompt lengths.

*Ɣ* is considered as an optional component in vanilla ICL that maps original classes (such as positive) *c* ∈ *C* to label words *v* ∈ *V* (such as “good,” “great,” or “wonderful”), as shown in Table 1. While *Ɣ* has not significantly contributed to in-domain LLM tasks, we empirically evaluate its contribution to OOD unseen tasks. Previous methods often relied on manual verbalizers, which could introduce biases, or automatic verbalizers that required explicit training to achieve better performance (Schick and Schütze, 2021; Liu et al., 2024). As illustrated in Figure 2, we use frozen ℳ as the tokenizer, *C*, *V*, and the OpenPrompt library to determine *Ɣ*. Here, *V* = {*v_1_*, *v_2_*, …, *v_n_*} identifies the depressive content, such as hurt, broken, and shocking, within *xq*. *Ɣ* is defined as a mapping function *f*, utilizing the LLM probability of each *v* being identified as a [MASK] token, to map content probabilities in *x* onto class probabilities of *p*(*c*|*x*) as shown in Equation 6.


(6) $$p(c|x)=f(p_{M}([\text{MASK}]=v|x)|v\in V)$$

**Table 1:** Sample of classes and label words.

Label	Normal	Depression
Label words	Joy, happy, elation, contentment	Hurt, anger, moody, bored, sadness

Finally, ℳ*_ꟷ_* integrates *P*, *D*, *E_rank_*, *₸*, and *Ɣ* to classify each *x* ∈ *xq* as “depressive” or “normal.” Consequently, we enhance the existing ProDepDet framework by incorporating *ExDoRA* to improve the reasoning of transferring the MPC modeling knowledge of an LLM ℳ to depression detection. Two specific hyper-parameters, *θ_1_* and *θ_2_*, are used to freeze ℳ and to disable its dropout, maintaining the fundamentals of ICL (Priyadarshana et al., 2024). The contextual embeddings of the classified *xq* are then processed through a non-linear transformation and normalized to facilitate the formation of downstream tasks. The results are shown in Table 2.

**Table 2:** Classification results.

Utterance	Label
Where have you been?	Normal
Oh! I wanted to *hurt* myself.	Depression
What happened to you?	Normal
I was *broken*, it’s *shocking*.	Depression

### 3.7 Downstream task formation

Two downstream tasks, Depressed Utterance Classification (DUC) and Depressed Speaker Identification (DSI), are formed considering the downstream tasks established for MPC modeling, including Reply Utterance Selection (RUS) and Speaker Identification (SI) (Lu et al., 2023; Priyadarshana et al., 2024). Here, we evaluate the transferability of the proposed framework using top-ranked explanations for previously unseen DUC and DSI. DUC, specialized from RUS, identifies specific *x* ∈ *xq* that are classified as containing depressive content *U_d_* and determines the exact speaker *S_d_*. This can be presented as Equation 7.


(7) $${\left\{(S_{d},U_{d})\right\}}_{n=1}^{\mathrm{xq}}\setminus \mathrm{xq}$$

The contextualized representations from the frozen model ℳ undergo a non-linear transformation to derive matching probabilities *U_DUC_* for the depressive context in *xq*. The loss value *L_DUC_*, related to the probability scores obtained and their ground truth labels, is calculated as Equation 8


(8) $$L_{\mathrm{DUC}}=-[z\;\log(U_{\mathrm{DUC}})+(1-z)\log(1-U_{\mathrm{DUC}})]$$

where ꟷ = 1 when *x* ∈ *xq* is an exact match for the depressive context and ꟷ = 0 otherwise.

DSI, specialized from SI, identifies the exact speaker *S_d_* of an utterance. Since the speakers vary across multiple utterances within *xq*, DSI is designed to determine the exact speaker shared by multiple utterances classified as depressed. The speaker embeddings derived from ℳ are further processed through a non-linear transformation layer and then normalized to obtain the matching probability values *U_DSI_* for depressive *x* ∈ *xq*. The cross-entropy loss *L_DSI_*, related to *U_DSI_* and the ground truth labels, is calculated as Equation 9


(9) $$L_{\mathrm{DSI}}=-\sum_{j=1}^{N-1}z_{i}.\log(U_{\mathrm{DSI}})$$

where ꟷ*_i_* = 1 when both *U_i_* and *U_j_* share the same speaker and ꟷ*_i_* = 0 otherwise.

## 4 Experiments

We conducted experiments to answer the following research questions.

*RQ1*: How does incorporating ERR- and MMR-based ranking improve both diversity and semantic relevance in ranking LLM-generated free-text explanations?

*RQ2*: How does leveraging free-text explanations of the retrieved ICL demonstrations contribute to LLM transferability for contextually different depression detection tasks?

*RQ3*: How do the number, order, and diversity of the top-ranked explanations enhance the robustness of the proposed framework?

### 4.1 Datasets

As shown in Table 3, the proposed methods for explanation generation and depression detection are evaluated on five benchmark datasets derived from MPC data, including human-annotated posts, comments, and chats from Twitter and Reddit. The IMHI corpus for the depression detection sub-task (Pirina and Çöltekin, 2018) is used to validate the generated top-ranked synthetic explanations. The RSDD corpus is used to generate *D* and evaluate the DUC task. Twitter Depression 2022 (Cha et al., 2022) is used to create classes and labels for *Ɣ* and evaluate DUC. For the DSI task, the eRisk 18 T2 (Losada et al., 2018) and eRisk 22 T2 (Losada et al., 2019) datasets are used, with a particular focus on capturing speaker characteristics in the context of MPC modeling. Table 4 shows a summary of depressed and normal speakers across these datasets.

**Table 3:** Statistical summary of datasets.

Benchmarks	Train	Validation	Test
Reddit IMHI Corpus 2024	1,003	430	405
Reddit SDD Corpus 2017	609,471	684,788	599,573
Reddit eRisk 18 T2 2018	49,557	20,332	20,333
Reddit eRisk 22 T2 2022	40,242	32,264	35,332
Twitter Depression 2022	35,586	15,000	15,000

**Table 4:** Statistical summary of users and posts.

Benchmarks	# User		# Post	
	Depressed	Normal	Depressed	Normal
Reddit SDD Corpus 2017	9, 000	107,000	920,184	960,487
Reddit eRisk 18 T2 2018	134	354	25,138	64,274
Reddit eRisk 22 T2 2022	98	658	35,332	153,436
Twitter Depression 2022	38	2,457	30,497	35,089

To ensure consistency, data balancing techniques, such as NearMiss (Jeon and Lim, 2020) undersampling, were employed.

### 4.2 Baselines

We used 7B-parameter LLMs, such as Mistral-7B-Instruct, Gemma-7B (Team et al., 2024), LLaMA-2-7B-chat (Touvron et al., 2023), and MentaLLaMA-chat-7B (Yang et al., 2024), to evaluate the explanation generation capabilities of *ExDoRA*. These models excel at generating detailed, relevant, and context-sensitive free-text explanations and are well-suited for processing long-form, multi-turn MPC data while simulating model reasoning processes. To evaluate explanation ranking, we used several similarity models: *all-MiniLM-L6-v2*, optimized for processing longer MPCs in large corpora without performance bottlenecks; *all-mpnet-base-v2*, adept at detecting subtle context shifts between different speakers; and *all-distilroberta-v1*, fine-tuned to capture semantic continuity and identify speaker roles across conversation threads against similar ranking methods, including *EGLR* by Liu et al. (2024), *ExplRank* by Ye and Durrett (2022), and a GPT-3-based method by Wiegreffe et al. (2021).

For the evaluations of DUC, we used WSW (Priyadarshana et al., 2023), MentalBERT (Ji et al., 2022), and DisorBERT (Aragon et al., 2023) as 100 M-300 M-parameter LLMs aware of MPC semantic modeling, with MentalBERT and DisorBERT being particularly used to detect mental disorders. For the evaluations of DSI, we used WSW, SA-BERT (Gu et al., 2020), and MPC-BERT (Gu et al., 2021) as speaker-aware MPC modeling LLMs. Additionally, LLaMA 2-7B (Touvron et al., 2023) and MentaLLaMA-7B (Yang et al., 2024) were used as open-source 7B-parameter LLMs and ChatGPT (OpenAI, 2022) and GPT-4 (Achiam et al., 2023) were adopted as 175B-1.76 T-parameter LLMs to evaluate both DUC and DSI.

### 4.3 Implementation details

The explanation ranking logic of *ExDoRA* and the OOD task transfer logic for depression detection were implemented using Python libraries, specifically PyTorch 2.0^3^ and Hugging Face Transformers.^4^ We used UDR^5^ as the demonstration retriever. We divided the MPC data into three categories based on session length, *Len-5*, *Len-10*, and *Len-15*, and experimented with two different prompt lengths (*l*) of 70 and 90. Two hyper-parameters, such as a maximum length of 3,000 and the number of generated explanations of 3, were used to generate explanations for each *D*. The lambda diversity and the decay factor were kept at 0.5 and 0.85, respectively, to obtain the top two explanations. We used other hyper-parameters for downstream tasks, such as GELU activations (Hendrycks and Gimpel, 2022) for non-linear transformations, Adam optimizer (Kingma, 2014) with a learning rate of 0.0005, a warmup proportion of 0.1, and enabled parameters *θ_1_* and *θ_2_*. The training was conducted over 30 epochs for 900 h (30 h per epoch) using dual NVIDIA RTX 3090 Ti 24GB GPUs with a batch size of 16. We used Application Programming Interface endpoints provided by OpenAI for evaluating closed-source LLMs. To ensure a fair comparison, all LLMs and similarity models used for explanation generation were evaluated under the same data and hyperparameter settings. *ExDoRA* has been made open-source to facilitate the replication of our results.^6^

### 4.4 Metrics and results

The generated explanations were evaluated based on two primary criteria: factuality and consistency. Factuality assessment focuses on ensuring that the generated explanations are contextually relevant and faithfully grounded. Inspired by Ye and Durrett (2022), we evaluated factuality using the lexical overlap between top-ranked explanations and ground truth explanations from IMHI. Considering *e_i_* as the candidate explanation and *s_g_* as the ground truth, the factuality estimation is defined as Equation 10.


(10) $$\text{factuality}(e_{i})=\max_{e_{i}}\frac{|e_{i}\cap s_{g}|}{|e_{i}|}$$

We used the top three explanations generated for 100 queries and validated them against the expert-written explanations from the IMHI corpus, sourced from multiple corpora, including DR and CAMS.^7^

**Figure 4:**
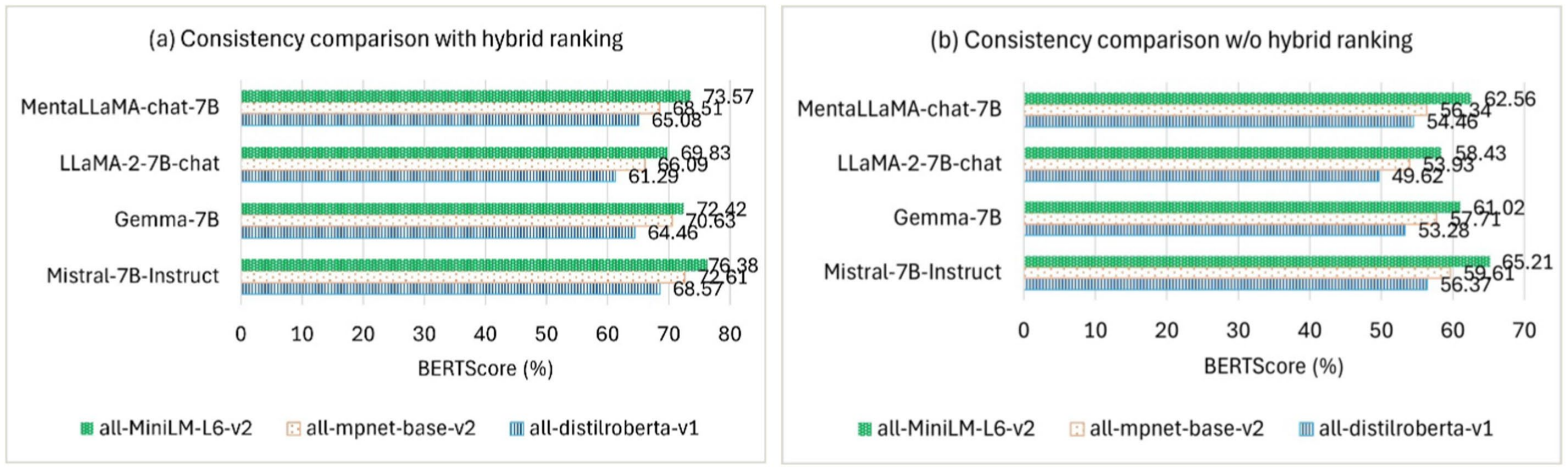
Consistency comparison in terms of BERTScore for three explanations.

To evaluate the consistency of the generated explanations, we used BERTScore (Zhang et al., 2020) to assess how well the explanations align with consistent reasoning across various examples, building on Ye et al. (2023), who empirically demonstrated the impact of LLM-generated explanations on downstream performance. The consistency is reformulated in terms of an alignment between the demonstration sample, its ground truth label, and the generated explanation. We selected 200 human-annotated depression samples from the RSDD corpus and then generated explanations to evaluate the consistency. Factuality and consistency together ensure that the generated explanations align with the context and contribute to the model’s reasoning for the final prediction. Table 5 shows the factuality comparison of the explanations using multiple similarity models against explanation ranking methods. Figure 4 shows the consistency comparison Figure 4a with and Figure 4b without (w/o) the proposed hybrid ranking.

**Table 5:** Results on the factuality of the generated explanations in terms of lexical overlap (%).

↓ LLM / Ranking method →	EGLR	ExplRank	GPT-3-based	ERR-MMR
All-distilroberta-v1 as ℳ_S_				
Mistral-7B-Instruct	**40.21** ± 0.16	**47.12** ± 0.12	52.14 ± 0.13	**61.27** ± 0.13
Gemma-7B	39.34 ± 0.21	43.17 ± 0.12	**54.28** ± 0.11	57.31 ± 0.21
LLaMA-2-7B-chat	37.24 ± 0.12	41.37 ± 0.14	52.19 ± 0.17	52.73 ± 0.22
MentaLLaMA-chat-7B	38.53 ± 0.23	42.28 ± 0.12	53.07 ± 0.12	54.71 ± 0.12
All-mpnet-base-v2 as ℳ_S_				
Mistral-7B-Instruct	**39.08** ± 0.13	**43.58** ± 0.21	**49.71** ± 0.11	**55.81** ± 0.16
Gemma-7B	38.31 ± 0.17	41.16 ± 0.22	48.83 ± 0.12	52.37 ± 0.21
LLaMA-2-7B-chat	36.47 ± 0.21	38.23 ± 0.11	45.37 ± 0.12	49.93 ± 0.17
MentaLLaMA-chat-7B	37.38 ± 0.13	39.14 ± 0.21	46.57 ± 0.21	50.07 ± 0.24
All-MiniLM-L6-v2 as ℳ_S_				
Mistral-7B-Instruct	**44.51** ± 0.21	**49.62** ± 0.34	**56.81** ± 0.33	**64.23** ± 0.31
Gemma-7B	42.41 ± 0.32	46.37 ± 0.22	53.27 ± 0.31	59.34 ± 0.28
LLaMA-2-7B-chat	39.71 ± 0.24	43.82 ± 0.17	51.21 ± 0.28	56.72 ± 0.17
MentaLLaMA-chat-7B	41.52 ± 0.18	44.27 ± 0.23	53.13 ± 0.21	58.21 ± 0.26

SOTA performance is shown in bold.

To evaluate the DUC task, we used R_10_@1, an enhanced recall metric commonly applied in SOTA models for MPC-based response selection tasks, such as RUS. This metric measures the rate at which the first correctly classified depressed utterances are identified among 10 candidates from RSDD and Twitter Depression 2022 corpora. Table 6 shows the results of the LLM’s OOD transferability for DUC using zero-shot (ZS), 2-shot (2S), and 5-shot (5S) D, both with and without (w/o) the top two explanations. To evaluate DSI, the F1 score was selected as the metric for the eRisk 18 T2 and eRisk 22 T2 benchmarks. Table 7 shows LLM OOD transferability results using ZS, 2S, and 5S D, both with and without (w/o) the top two explanations.

**Table 6:** Evaluation results of DUC.

Setting	Model	RSDD Corpus						Twitter Depression 2022 Corpus					
		*Len-5*		*Len-10*		*Len-15*		*Len-5*		*Len-10*		*Len-15*	
		*l* = 70	*l* = 90	*l* = 70	*l* = 90	*l* = 70	*l* = 90	*l* = 70	*l* = 90	*l* = 70	*l* = 90	*l* = 70	*l* = 90
ZS D (w/o E)	MentalBERT	70.04	71.24	67.81	69.62	62.83	64.41	67.81	68.54	65.07	67.93	64.59	65.97
	DisorBERT	68.73	69.93	65.48	65.02	63.27	64.11	66.83	68.07	64.37	66.71	64.13	65.86
	WSW	72.61	74.08	69.37	70.14	65.81	67.08	71.03	72.95	69.02	71.87	66.81	68.01
	LLaMA 2-7B	69.02	71.27	68.83	70.09	64.04	66.19	70.09	72.04	68.27	70.24	65.19	67.14
	MentaLLaMA	**72.86**	**74.27**	**70.08**	**71.38**	**66.29**	**68.64**	**72.26**	**73.57**	**70.11**	**72.27**	**68.18**	**70.07**
	ChatGPT	55.21	57.12	53.29	54.83	52.04	53.87	49.72	51.04	48.01	50.27	46.87	48.31
	GPT-4	59.34	62.28	58.06	60.17	55.67	57.34	57.81	59.37	55.82	57.08	54.05	56.27
2S D (with E)	MentalBERT	84.67	85.49	80.37	82.93	79.67	81.63	81.69	83.61	80.37	81.64	79.67	80.38
	DisorBERT	83.06	84.69	81.64	82.69	79.33	81.17	81.08	82.68	79.61	80.37	78.05	79.61
	WSW	**87.28**	**88.93**	**84.06**	**86.28**	**82.27**	**84.07**	**84.67**	**86.08**	**82.46**	**84.06**	**81.93**	**82.28**
	LLaMA 2-7B	82.27	83.97	81.06	82.37	79.67	81.36	80.39	82.64	79.38	81.28	78.19	80.34
	MentaLLaMA	84.93	86.73	82.48	84.61	80.35	82.97	82.67	84.05	80.88	82.06	80.24	81.09
	ChatGPT	72.04	74.43	70.11	72.27	69.91	70.03	68.81	70.26	66.30	68.03	64.55	66.21
	GPT-4	74.39	76.68	72.13	74.44	70.12	72.61	72.39	74.47	70.29	72.34	68.51	70.63
2S D (w/o E)	WSW	85.73	87.49	82.64	84.08	80.39	81.72	82.93	84.76	80.34	82.07	79.77	80.21
	MentaLLaMA	82.53	84.67	81.87	83.73	79.61	81.89	81.76	83.54	79.82	81.94	79.37	80.28
	GPT-4	72.03	74.88	70.63	69.51	68.51	69.21	69.47	71.31	67.54	68.91	65.04	67.53
5S D (with E)	MentalBERT	90.52	92.24	89.61	91.16	87.89	89.31	89.92	90.28	87.35	88.68	85.61	86.53
	DisorBERT	90.67	91.28	88.62	89.32	86.62	87.93	89.21	90.01	88.28	89.65	86.24	87.68
	WSW	**92.57**	**94.67**	**90.55**	**92.64**	**88.59**	**90.51**	**91.21**	**93.17**	**89.64**	**91.34**	**87.62**	**89.52**
	LLaMA 2-7B	89.03	90.56	87.38	88.55	85.57	86.69	87.08	88.61	85.61	86.39	83.81	85.28
	MentaLLaMA	91.28	93.67	89.80	91.28	87.96	89.62	90.08	91.37	88.76	90.21	86.57	88.09
	ChatGPT	81.28	83.24	79.51	82.27	77.81	79.20	78.27	80.11	76.59	78.21	75.12	76.58
	GPT-4	86.91	89.14	85.27	88.16	83.21	86.34	84.18	86.34	82.61	84.63	81.37	83.62
5S D (w/o E)	WSW	91.24	92.84	89.31	91.22	86.21	88.51	89.01	91.26	87.61	88.61	85.72	87.31
	MentaLLaMA	88.97	89.76	87.89	89.73	85.97	87.29	88.94	90.73	87.04	88.13	84.39	86.73
	GPT-4	84.52	86.81	83.46	84.24	81.34	82.51	82.84	84.26	80.24	82.44	79.92	81.31

SOTA performance is shown in bold. The second-best performance is shown with underlining. ZS, 2S, and 5S stand for zero-shot, 2-shot, and 5-shot, respectively; *D* and *E* stand for demonstrations and explanations.

**Table 7:** Evaluation results of DSI.

Setting	Model	Reddit eRisk 18 T2 Corpus						Reddit eRisk 22 T2 Corpus					
		*Len-5*		*Len-10*		*Len-15*		*Len-5*		*Len-10*		*Len-15*	
		*l* = 70	*l* = 90	*l* = 70	*l* = 90	*l* = 70	*l* = 90	*l* = 70	*l* = 90	*l* = 70	*l* = 90	*l* = 70	*l* = 90
ZS D (w/o E)	SA-BERT	59.18	63.27	58.31	61.63	56.34	59.61	54.73	57.76	52.08	54.63	50.33	52.74
	MPC-BERT	63.52	65.61	61.04	63.37	59.48	61.21	58.61	60.27	56.83	58.81	55.07	57.96
	WSW	**65.89**	**67.04**	**62.84**	**64.34**	**59.97**	**62.27**	**61.05**	**63.26**	**59.34**	**61.11**	**57.89**	**59.06**
	LLaMA 2-7B	58.27	60.01	55.59	57.04	53.59	55.04	52.24	54.49	50.07	52.67	48.97	50.14
	MentaLLaMA	62.28	64.33	60.04	62.57	58.67	60.31	57.89	59.34	55.27	57.75	53.04	55.79
	ChatGPT	46.58	48.21	43.58	45.37	40.27	42.26	44.71	46.92	42.58	44.24	39.61	41.16
	GPT-4	49.67	52.24	45.59	47.47	42.64	44.07	46.84	48.39	44.91	46.37	41.38	43.64
2S D (with E)	SA-BERT	76.27	78.28	74.61	76.68	72.31	74.06	72.81	74.28	70.06	72.34	68.34	70.13
	MPC-BERT	79.02	81.24	77.62	79.28	75.53	77.01	76.34	78.19	74.62	76.29	72.61	74.03
	WSW	**82.06**	**84.52**	**80.17**	**82.26**	**78.61**	**80.31**	**79.61**	**81.61**	**77.64**	**79.34**	**75.24**	**77.18**
	LLaMA 2-7B	77.28	79.34	75.06	77.29	73.61	75.39	73.28	75.61	71.05	73.64	69.28	70.38
	MentaLLaMA	80.38	82.34	78.59	80.14	76.64	78.67	77.82	79.64	75.94	77.51	73.39	75.06
	ChatGPT	66.27	68.61	64.59	66.07	62.37	64.58	62.04	64.37	60.72	62.46	58.22	59.67
	GPT-4	72.57	74.46	70.06	72.31	68.15	70.34	68.34	70.16	66.91	68.32	64.43	66.33
2S D (w/o E)	WSW	79.05	81.26	76.61	78.11	73.25	75.21	75.37	77.51	72.27	74.61	70.11	72.06
	MentaLLaMA	76.27	78.12	74.06	76.34	72.18	74.34	72.34	74.58	70.31	72.19	68.22	70.32
	GPT-4	68.24	70.06	66.37	68.31	64.72	66.32	63.28	65.66	61.34	63.61	59.82	61.04
5S D (with E)	SA-BERT	79.34	81.04	77.64	79.81	75.32	76.68	75.57	77.08	72.92	75.38	71.05	73.64
	MPC-BERT	82.26	84.05	80.06	82.11	78.09	80.24	78.61	80.62	76.24	78.16	74.28	76.08
	WSW	**86.62**	**88.62**	**84.59**	**86.31**	**82.66**	**84.51**	**82.27**	**84.05**	**80.22**	**82.36**	**78.61**	**80.61**
	LLaMA 2-7B	80.36	82.64	78.68	80.15	76.64	78.37	77.62	79.38	75.25	77.68	73.91	75.57
	MentaLLaMA	84.68	86.24	82.14	84.35	80.06	82.11	79.89	81.24	77.67	79.34	75.69	77.51
	ChatGPT	76.37	77.83	74.55	76.06	72.01	74.68	72.25	74.64	70.06	72.64	68.83	70.23
	GPT-4	81.05	83.68	79.64	81.05	77.69	79.34	77.68	79.73	75.25	77.61	73.61	75.38
5S D (w/o E)	WSW	84.36	86.62	82.19	84.67	80.67	81.29	79.68	81.16	77.25	79.61	75.61	77.62
	MentaLLaMA	82.21	84.59	80.11	82.64	78.93	79.21	76.39	78.21	74.59	76.31	72.83	74.39
	GPT-4	78.28	80.34	76.59	78.37	74.32	76.11	74.24	76.13	72.28	74.05	70.37	72.13

SOTA performance is shown in bold. The second-best performance is shown with underlining. ZS, 2S, and 5S stand for zero-shot, 2-shot, and 5-shot, respectively; *D* and *E* stand for demonstrations and explanations.

### 4.5 Ablation studies

A series of ablation studies were conducted on random 5S *D* splits of the Twitter Depression corpus and eRisk 22 T2 corpus to validate the generalizability of the proposed methods. These studies focused on evaluating the number, order, and diversity of top-ranked explanations with and without (w/o) the hybrid ranking component of *ExDoRA*.

**Figure 5:**
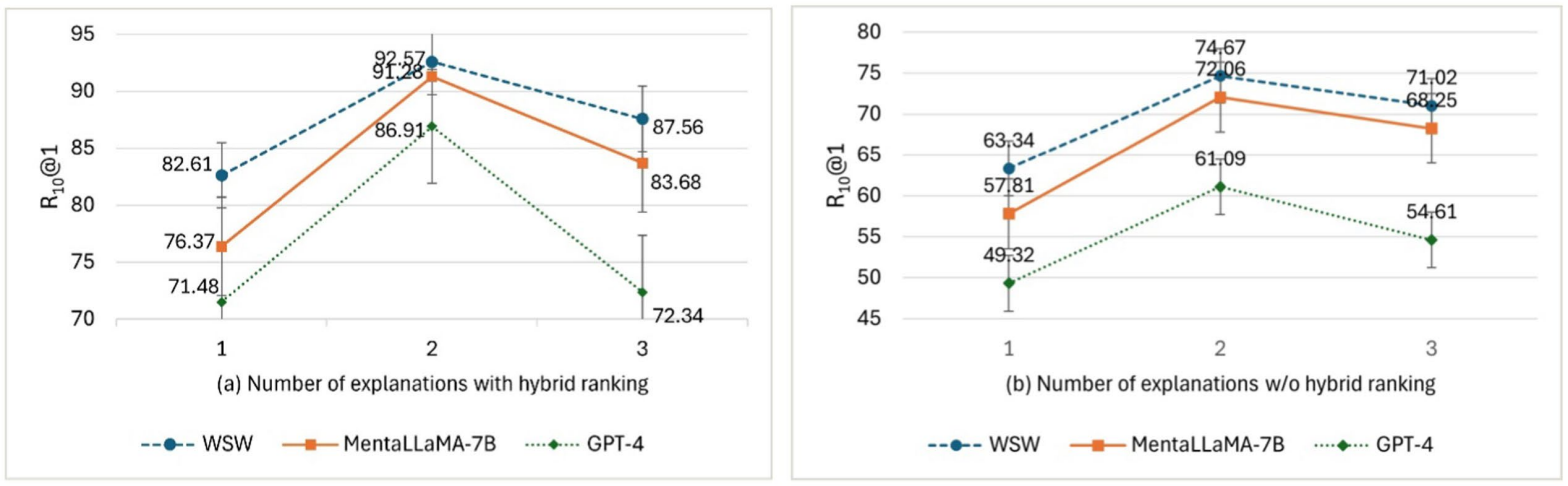
The effect of the number of top-ranked explanations with and w/o the hybrid ranking.

The number of top-ranked explanations from the Twitter Depression corpus was used to compare *ExDoRA*’s performance in DUC. Figure 5 shows the evaluation results of the best-performing LLMs for DUC in terms of R_10_@1 using the top-1, top-2, and top-3 ranked explanations.

**Figure 6:**
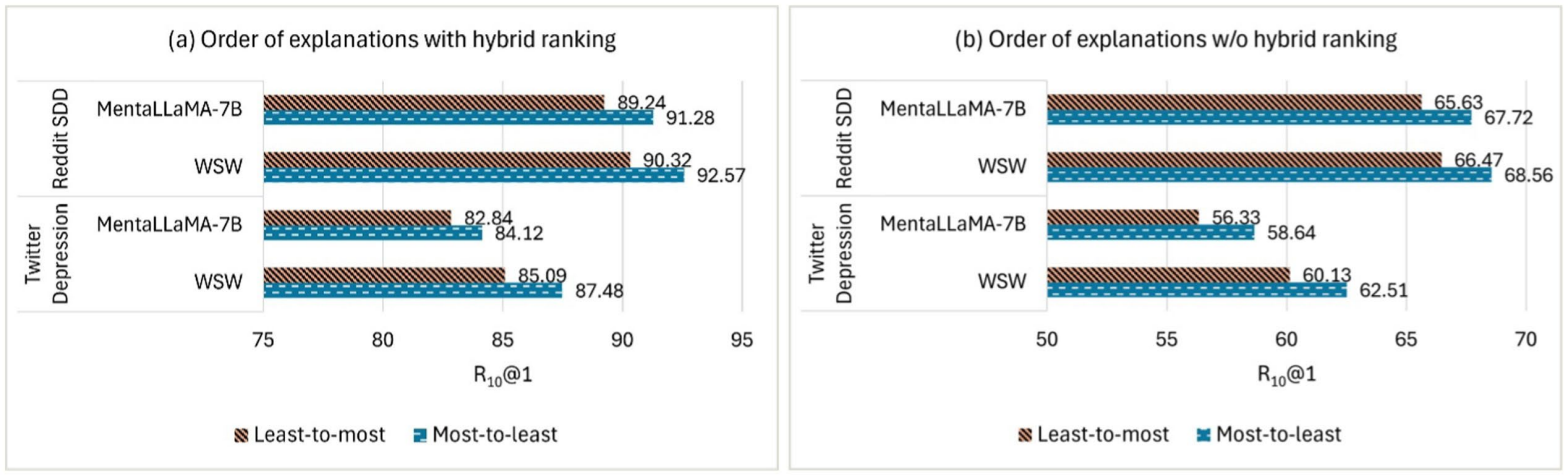
The effect of the order of top-ranked explanations with and w/o the hybrid ranking.

The order of top-ranked explanations from the RSDD and Twitter Depression 2022 corpora was used to evaluate *ExDoRA*’s performance. Figure 6 shows the evaluation results for the best-performing LLMs in DUC in terms of R_10_@1 using the least-to-most and most-to-least ordering of the top three explanations. The most-to-least prioritizes explanations with the highest semantic relevance, placing them at the beginning, while the least-to-most ordering positions the least relevant explanations first.

**Figure 7:**
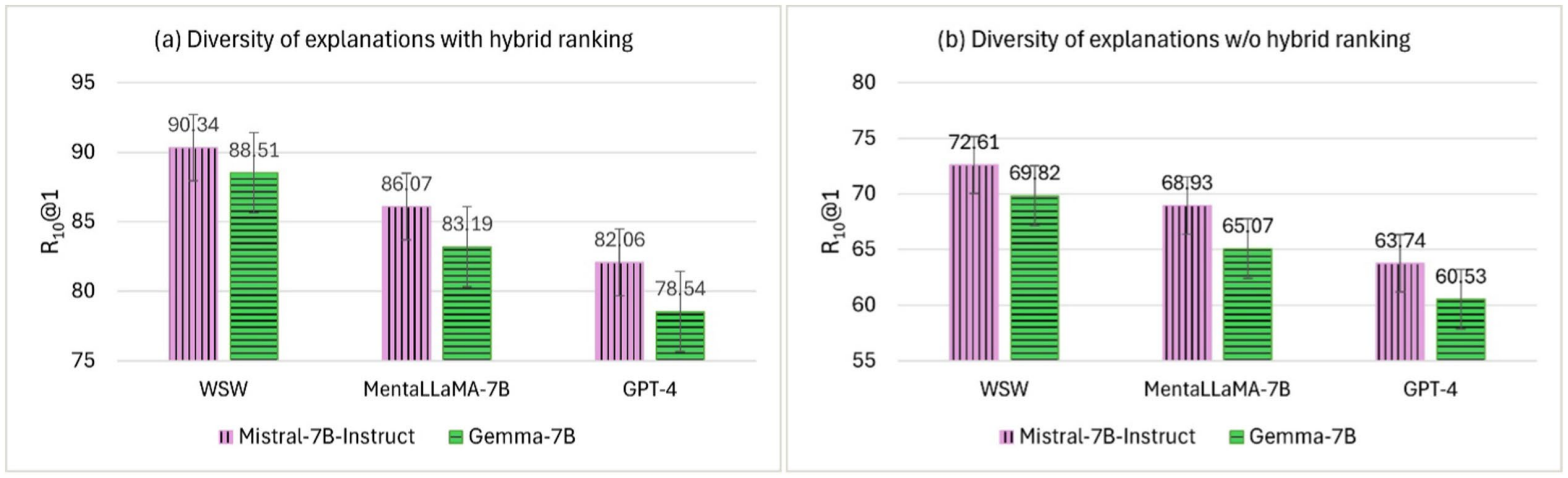
The effect of the diversity of top-ranked explanations with and w/o the hybrid ranking.

The diversity of top-ranked explanations from the RSDD corpus was used to compare *ExDoRA*’s performance in DUC. Two sets of *E_rank_* were generated using Mistral-7B-Instruct and Gemma-7B LLMs to create a diverse range of the top three explanations. Figure 7 shows the evaluation results of the best-performing LLMs in DUC in terms of R_10_@1 using the two different explanation sets.

## 5 Discussion

### 5.1 *ExDoRA* performance

The evaluation results in Table 5 indicate that the combination of Mistral-7B-Instruct with all-MiniLM-L6-v2 effectively generates highly factual free-text explanations using the proposed ERR- and MMR-based ranking method. Mistral-7B-Instruct, fine-tuned on instruction-based data to enhance understanding of nuanced text features, outperformed other open-source LLMs in generating the most appropriate and context-sensitive free-text explanations, particularly when paired with all-MiniLM-L6-v2 as ℳ*_S_*. The generated explanations are then ranked using multiple ranking methods, and the results show that our proposed ERR- and MMR-based methods perform well compared to other alternative ranking methods. Particularly, Mistral-7B-Instruct with all-MiniLM-L6-v2 demonstrated significant performance improvements, achieving 8.42 and 2.96% gains in lexical overlap when ranking explanations using the ERR- and MMR-based ranking method, compared to all-mpnet-base-v2 and all-distilroberta-v1. This highlights the superiority of both ℳ*_S_* and the proposed ranking method in processing longer MPCs within large corpora. The method accounts for both diversity and semantic relevance in ranking multiple generated explanations while avoiding performance bottlenecks. Therefore, RQ1—*How does incorporating ERR- and MMR-based ranking improve both diversity and semantic relevance in ranking LLM-generated free-text explanations?*—can be considered answered. Figure 4a shows that Mistral-7B-Instruct outperformed other open-source LLMs paired with all-MiniLM-L6-v2 by a maximum margin of 2.81% in BERTScore, aligning the top three generated explanations with consistent reasoning across various examples. However, the performance dropped substantially by up to 11.17% in BERTScore, as shown in Figure 4b, when the top-ranked explanations were excluded.

### 5.2 Performance on DUC and DSI

Experiments on DUC and DSI showed that *ExDoRA* enhanced the adaptation of acquired knowledge of the source LLM to modeling contextually different depression detection tasks by leveraging the free-text explanations of the retrieved ICL demonstrations. Table 6 shows that WSW, the SOTA LLM for MPC modeling, outperformed both open-source and closed-source LLMs for DUC in 2S and 5S demonstration settings. While MentaLLaMA-7B, the second best-performing LLM, showed some effectiveness without demonstrations and explanations in certain test cases, WSW achieved SOTA performance in DUC when demonstrations and their top-ranked explanations were available. This highlights *ExDoRA*’s effectiveness in transferring MPC modeling knowledge of LLMs for depression detection. The performance of WSW in DUC was improved by significant margins of 20.59 and 20.22% in terms of R_10_@1 when using 5S demonstrations with the top two explanations, compared to ZS examples without explanations for MPC data split with *Len-5* and *l* = 90 in RSDD and Twitter Depression 2022 corpora. However, the performance slightly dropped by margins of up to 1.44 and 1.83% in terms of R_10_@1 when using 2S and 5S demonstrations without the top-ranked explanations, highlighting the importance of *ExDoRA* for LLM OOD knowledge transfer. The overall performance marginally dropped as the MPC session length increased, although longer prompt lengths contributed to integrating richer contextual representations with demonstrations and explanations. The ZS demonstration performance of LLMs such as GPT-4 fell short compared to the 100 M-300 M-parameter LLMs due to the absence of explicit information related to MPC understanding. However, LLM performance on DUC improved to near-SOTA levels with the inclusion of MPC explicit data in 2S and 5S demonstration settings with the top two explanations.

For the DSI task, determining the exact speaker of an utterance classified as depressed presents a challenge when speaker details are not available. Table 7 shows that WSW performed better than both the clinical-based LLMs, such as MentaLLaMA-7B, and generative LLMs, such as GPT-4, in shifting MPC speaker identification to DSI due to the lack of explicit speaker information in those LLMs. The performance of WSW for DSI significantly improved by margins of 21.58 and 20.79% in terms of F1 score using 5S demonstrations with the top two explanations, compared to ZS demonstrations without explanations for MPC data split with *Len-5* and *l* = 90 in eRisk 18 T2 and eRisk 22 T2 corpora. This is because speaker-aware MPC modeling LLMs, including SA-BERT, MPC-BERT, and WSW, can incorporate implicit speaker details into MPC discourse structures of which other LLMs are not aware. WSW, in particular, is the SOTA LLM to process speaker details in complex discourse structures, such as root-level and sub-level utterances classified as depressed. Conversely, the performance slightly dropped by maximum margins of 3.26 and 2% in terms of F1 score when using 2S and 5S examples, respectively, without the top-ranked explanations, highlighting the significance of *ExDoRA*’s *reason-then-predict* approach for previously unseen DSI. Compared to DUC, the ZS performance of LLMs was inferior to that of 100 M-300 M-parameter LLMs, such as WSW and MPC-BERT, for speaker identification in MPC modeling due to the absence of explicit speaker information. However, LLM performance on DSI improved to near-SOTA levels with MPC explicit data in 2S and 5S demonstration settings with the top-ranked explanations. Although the overall DSI performance slightly dropped with increased MPC session length, it improved with increased prompt length, leading to WSW, MentaLLaMA-7B, and GPT-4 being the top performers. It can be concluded that selecting in-context demonstrations with their top-ranked explanations for few-shot prompting offers SOTA performance in OOD tasks. Therefore, RQ2—*How does leveraging free-text explanations of the retrieved ICL demonstrations contribute to LLM transferability for contextually different depression detection tasks?*—can be considered answered.

### 5.3 Impact of ablation studies

We conducted several ablation studies using the best-performing models in 100 M-300 M-parameter LLMs, open-source 7B-parameter LLMs, and closed-source 175B-1.76 T-parameter LLMs for DUC and DSI. The results on the effect of the number of top-ranked explanations, presented in Figures 5a,b, revealed that the performance of DUC significantly decreased by margins of 16.54, 15.43, and 17.73% in terms of R_10_@1 when using three explanations for WSW, MentaLLaMA-7B, and GPT-4, after removing the hybrid ranking component of *ExDoRA*. This decline can be attributed to the diminished quality of the selected demonstrations and the reduced reasoning capability of LLMs for depression detection tasks when their accompanying explanations were absent. Furthermore, we observed that the performance of DUC decreased as the number of ranked explanations increased beyond the top two. This reduction in performance can be attributed to the fact that while the number of contextual representations of demonstrations and the top explanations for the previously unseen depression detection tasks increased, the LLMs’ ability to capture and integrate this clinical context with the contextual representations of the MPC modeling task diminished due to scalability issues.

Moreover, we conducted a few ablation studies on the order of the top-ranked explanations. Figure 6 reveals that the LLMs performed better for DUC with the most-to-least ordering of explanations on the Twitter Depression 2022 corpus, whereas the opposite was observed on the RSDD corpus. Similar to the behavior for the number of explanations, the performance of WSW for DUC significantly decreased by margins of 24.97 and 24.01% in terms of R_10_@1 for the most-to-least ordering of explanations on Twitter Depression 2022 and RSDD corpus, respectively, after removing the hybrid ranking component. Despite some improvements with the least-to-most ordering on the RSDD corpus, these gains were smaller compared to the most-to-least ordering on Twitter Depression 2022. This indicates that the order of explanations is data-dependent, and the most-to-least ordering contributes more significantly to *ExDoRA*’s performance.

The diversity of explanations is rarely explored, particularly in ICL-based in-domain task transfer, largely due to its complexity (Luo et al., 2024). Having a variety of explanations ensures that the model captures different reasoning paths, offering multiple perspectives on the same depression cues while reducing the risk of overfitting specific examples. Figure 7 shows that LLMs performed notably well for DUC when using the free-text explanations generated by Mistral-7B-Instruct as the benchmark for the diversity of demonstrations over the explanations generated by Gemma-7B. However, compared to Figures 7a,b, the downstream performance of WSW for DUC significantly decreased after removing the hybrid ranking component, with reductions of 17.73 and 18.69% in terms of R_10_@1 for explanations generated by Mistral-7B-Instruct and Gemma-7B, respectively. This decline can be attributed to the high-quality explanations generated by Mistral-7B-Instruct, when paired with all-MiniLM-L6-v2, contributing more significantly to downstream performance than other alternatives. The broader coverage of reasoning patterns through a variety of explanations enhances overall downstream performance, as demonstrated by evaluations where the explanations were ranked by relevance, thereby improving transferability without compromising predictive accuracy. Therefore, RQ3—*How do the number, order, and diversity of the top-ranked explanations enhance the robustness of the proposed framework?*—can be considered answered.

### 5.4 Challenges, ethical considerations, and limitations

There are a few challenges and limitations to our approach. A significant challenge is mitigating LLM bias in generating and selecting the top-ranked explanations. Although we applied several techniques such as ERR- and MMR-based ranking and cross-entropy loss to reduce LLM bias in the preference of explanations, careful attention must be paid to the source prompts and the selected in-context examples. To address bias in social media data, strategies such as data augmentation and adversarial debiasing can help. Data augmentation techniques, such as synonym replacement and style transfer, can introduce variation when generating synthetic explanations. In addition, incorporating an adversarial network to detect and mitigate bias in the generated explanations can improve the balance, accuracy, and generalizability of both the explanation generation and the downstream depression classification across different groups and contexts. Incorporating datasets from a wide variety of sources other than Twitter and Reddit will better capture population diversity. Expert evaluations, including human-in-the-loop feedback and reinforcement learning from human feedback, can further ensure unbiased explanation selection, model generalization, and robustness. Additionally, employing data anonymization is crucial to avoid confidentiality and accountability issues. Another significant challenge is preventing overfitting due to biases inherent in soft prompts and verbalizers, which must be managed to avoid scalability issues. Carefully designed mixed prompt templates that combine both soft and manual templates may help mitigate overfitting. However, this approach falls outside the scope of the current study and still requires validation through empirical testing and expert review. Avoiding manipulations that lead LLMs to generate erroneous results in depression classification using crafted prompts remains a difficult task. Although *ExDoRA* enhances transferability for OOD few-shot prompting, the differences between LLM reasoning mechanisms and human learning in adapting knowledge to new tasks require further exploration.

Incorporating LLM-generated explanations to work with sensitive mental health data raises several ethical concerns that must be carefully addressed. Bias and fairness in LLMs may result in biased explanations if the in-context examples reflect stereotypes, impacting vulnerable groups. Despite their advanced natural language understanding for mental health screening, LLMs, like other models, are trained on vast amounts of human-generated content and inherently reflect human biases. Models trained on MPC data risk inadvertent privacy violations if user content is not anonymized. It is important to handle mental health-related data with care and ensure anonymity in future analyses. LLMs process sensitive user data, such as personal conversations and mental health disclosures, which poses risks related to data breaches and misuse. Without robust privacy measures, including data encryption, secure storage, and access controls, confidential information could be exposed, leading to potential harm to individuals. Accountability is another key concern. If an LLM-generated explanation or classification leads to inaccurate mental health assessments or inappropriate recommendations, determining responsibility becomes difficult, especially when the model operates as a “black-box.” Carefully designed prompt templates and verbalizers should be used to mitigate uncertainty, user accountability, and confidentiality-related issues in ICL-based depression detection tasks. Accountability and explainability demand that explanations align with clinical standards to avoid misleading healthcare decisions. The psychological effects and clinical relevance should be thoughtfully evaluated to determine the quality of generated explanations, in-context examples, and prompt designs utilized in downstream tasks.

The present study is limited to generating free-text explanations, and the proposed ranking needs to be evaluated alongside explanations generated by other techniques, such as structured explanations. Although we limited the demonstration retriever to UDR due to its effectiveness in retrieving demonstrations from unseen datasets across multiple ICL tasks, incorporating other retrieval techniques could improve the system’s robustness. The validation of *ExDoRA* was conducted using the IMHI corpus, which is currently the only dataset for interpretable mental health analysis in social media, highlighting a significant limitation. To ensure the generalizability of our findings, additional evaluations should be conducted on relevant benchmarks in other fields, including in the clinical domain. Additionally, the generated free-text explanations should be externally validated by human experts, incorporating their feedback to enhance plausibility and informativeness, as our study relied solely on automatic evaluations. In this study, we focused on transferring LLM’s knowledge of MPC modeling to depression detection as a *reason-then-predict* approach. Future evaluations should explore alternative methods, such as *predict-then-reason* techniques like chain-of-thought reasoning, which may yield more promising results. Although task-specific instructions are critical for certain few-shot reasoning tasks, this study did not consider such instructions alongside in-context examples and their explanations. Our approach was limited to open-source 7B-parameter foundational LLMs for explanation generation. Although LLM quantization enables hosting much larger models, we restricted its use to avoid vulnerabilities such as jailbreaking and prompt injection. Further evaluations should include larger models, such as LLaMA-2-70B and LLaMA 3, with greater computational resources to assess how the proposed methods improve performance with increased scale. Furthermore, designing multiple soft prompt templates and verbalizers tailored to the characteristics of the target task could potentially impact the scalability of the proposed methods.

## 6 Conclusion and future research

In this article, we proposed *ExDoRA*, a novel framework designed to identify the most appropriate in-context examples using free-text explanations for depression detection in MPC data using LLM OOD task transferability. An ERR- and MMR-based hybrid method was introduced as the key contribution of the study, designed to rank LLM-generated explanations by selecting the most semantically relevant in-context examples closest to the input MPC query while balancing diversity and semantic relevance. To achieve the previously unseen depression detection tasks, we combined the in-context examples and their explanations from unseen data with soft embeddings of MPC input prompts using soft prompt templates and verbalizers. Evaluations on the IMHI corpus showed that *ExDoRA* generates highly factual and consistent free-text explanations. Extensive experiments were conducted using multiple LLMs for downstream tasks, including depressed utterance classification and depressed speaker identification. Evaluation results, including ablation studies, demonstrated that *ExDoRA* achieves SOTA performance in LLM OOD knowledge transfer for depression detection by leveraging in-context explanations.

Employing reinforcement learning agents to enhance user interactivity presents a promising avenue for ensuring unbiased, interpretable explanation selection by refining the process as clinician-in-the-loop and enhancing LLM generalization. Improving the domain-specific knowledge of *ExDoRA* with large-scale interpretable mental health corpora could further enhance the generalization of our methods across diverse domains. Generating synthetic explanations based on medical history and lifestyle data for disease prediction would further validate the present findings and contribute to developing universal clinical decision support systems. Our future studies will extend this framework to develop a multi-modal screening tool for depression detection in MPC data, integrating emotion-based approaches. Designing various downstream tasks that utilize prompt intelligence and automation is an encouraging direction to further improve the interpretability and scalability of LLMs, potentially addressing a wider array of mental health issues.

## Figures and Tables

**Figure A1:**
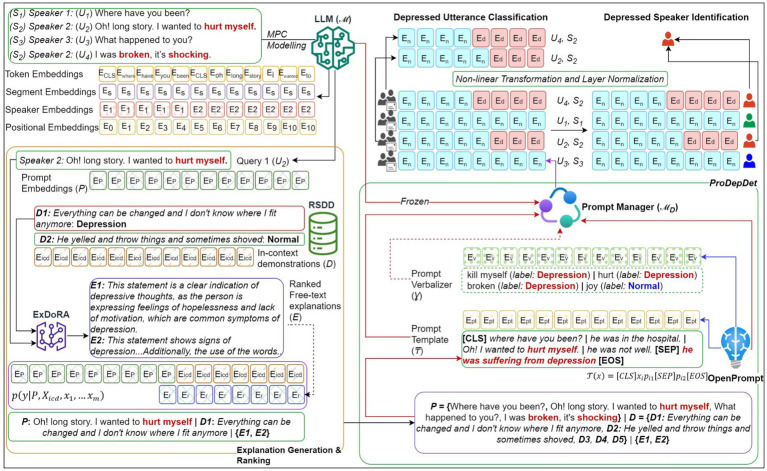
The LLM, with its knowledge of processing contextualized representations of MPCs, produces contextual embeddings for each utterance in the MPC query. The demonstration retriever selects the most semantically relevant demonstrations from the RSDD depression corpus that are closest to the input MPC query. These demonstrations are used to generate and rank free-text explanations for the query 1, U2, using ExDoRA in the Explanation Generation and Ranking phase. Once the prompt embeddings P are paired with the demonstrations and explanations, the prompt manager of ProDepDet processes the embedded P using mandatory prompt templates and optional verbalizers created from OpenPrompt Python library for downstream depression detection, including depressed utterance classification and depressed speaker identification.

## Data Availability

The original contributions presented in the study are included in the article/supplementary material, further inquiries can be directed to the corresponding author.

## Footnotes

^1^ https://huggingface.co/sentence-transformers

^2^ https://huggingface.co/sentence-transformers/all-MiniLM-L6-v2

^3^ https://pytorch.org/

^4^ https://github.com/huggingface/transformers

^5^ https://github.com/KaiLv69/UDR

^6^ https://github.com/KUAS-ubicomp-lab/ExDoRA

^7^ https://github.com/SteveKGYang/MentalLLaMA/tree/main?tab=readme-ov-file#expert-written-golden-explanations
